# Redesigning team-based learning facilitation for an online platform to deliver preclinical curriculum: A response to the COVID-19 pandemic

**DOI:** 10.15694/mep.2020.000135.1

**Published:** 2020-06-25

**Authors:** Peiyan Wong, Muhammad Raihan Jumat, Irene Cheng Jie Lee, Ke Xiang Foo, Suzanne Pei Lin Goh, Sashikumar Ganapathy, Siang Hui Lai, Nian Chih Hwang

**Affiliations:** 1 Duke-NUS Medical School; National University of Singapore; 2 Duke-NUS Medical School; 3 Duke-NUS Medical School; KK Women’s and Children Hospital; 4 Duke-NUS Medical School; Singapore General Hospital

**Keywords:** eLearning, Remote Learning, Flipped Classroom, Home-Based Learning

## Abstract

This article was migrated. The article was marked as recommended.

Due to the increasing number of COVID-19 cases globally, and the need for critical containment, Duke-NUS Medical School, Singapore, moved all of its preclinical classes online, keeping with national and university guidelines. The sudden move from face-to-face to online learning posed several challenges to the school’s team-based learning (TBL) pedagogy. In TBL, student engagement is key to promote peer-to-peer learning. The educational faculty found that it was challenging to ensure student engagement through an online platform. Additionally, online TBL is heavily dependent on the use of technology. Technological and internet connectivity issues were potential obstacles to the learning process. This manuscript proposes practical tips for a facilitator of an online TBL class to engage learners in this new format. To overcome technical complications, a dedicated centralized administrative team managed the logistics of hosting TBL online. Working synergistically, the facilitator, and the administrative team were instrumental in recreating the learning environment of a face-to-face TBL in an online platform.

## Introduction

Due to the increasing numbers of COVID-19 infections in Singapore, alert status was escalated and all learning institutions were closed on the 3 ^rd^ of April 2020 ( Mohan and Abu Baker, 2020; Mohan and Ang, 2020). In order to ensure academic continuity of our preclinical curriculum, the medical educators at Duke-NUS Medical School anticipated the restrictive measures and moved the learning onto an online platform in February 2020. The preclinical curriculum, which revolves around foundational scientific understanding, is delivered through a team-based learning (TBL) pedagogy, as has been described in detail elsewhere ( Michaelsen and Sweet, 2008; Parmelee *et al.*, 2012; Sibley and Ostafichuk, 2014). In brief, a typical TBL class starts before the in-class session, with advance preparation. For the first phase of TBL, students undergo the Readiness Assurance Test on learned material. These are a set of single-best multiple-choice questions that are attempted first individually, then as a group. The Group Assurance Test (GRAT) is designed to give immediate feedback. At Duke-NUS, immediately following GRAT, students have the opportunity to post queries to the class for the facilitated discussion session. In this class activity, which we term as modified TeamLEAD Readiness Assurance Process (mTRAP) ( Duke-NUS, 2011), students submit their team questions onto a common platform, and the facilitator then assigns to other teams to be answered. The facilitator moderates the class discussion, ensures high levels of engagement, and at the end of each discussion, invites the faculty to provide closure to the question ( Duke-NUS, 2011; Gullo, Ha and Cook, 2015). For the second phase of TBL, the students are assessed on their ability to apply the prior knowledge and concepts that they have mastered. In this phase, students work in their teams on a set of clinical scenario-based exercises. As the responses are entered online, the faculty have access to the answers as soon as they have been submitted. This phase is concluded with an in-class facilitated discussion, where the students are required to defend their team’s answers. At an appropriate juncture, the clinical content expert closes each discussion ( Duke-NUS, 2011). Alongside the facilitator is a team of administrators who manage the logistics of running a TBL module.

The rapid adaptation to online TBL uncovered several challenges to achieving the learning objectives of the preclinical phase. We faced two main obstacles when we moved the preclinical curriculum TBL classes online for our cohort of 82 students. After undergoing several iterations of online TBL, by incorporating student and faculty feedback, we came up with effective facilitation strategies that helped us overcome these challenges.

## The challenges we faced during the implementation of online TBL classes

Our first challenge was the difficulty in monitoring student engagement. The face-to-face interaction, which plays a large role in allowing the facilitator to detect non-verbal cues of the entire class at a glance ( Lane, 2008), is compromised in online TBL. Any online video-conferencing program has its limits on the maximum number of participants that could be viewed on a single screen, and we were unable to see the whole class at a glance. Even if the student is visible on the video-conferencing program, the ability to determine the student’s focused attention and full engagement remained elusive. This potentially undermines the interactive dynamics that is crucial for TBL. The lack of clear visibility on students’ learning behaviour could also challenge faculty’s ability to monitor student’s well-being and professionalism.

Our second challenge was that internet connectivity and technological literacy problems hindered smooth implementation of online TBL. Online TBL is heavily dependent on the use of multiple technologies and platforms. These include video-conferencing tools, test-taking platforms and a live shared document for students to discuss pertinent topics to the session. Participants (students and faculty) are expected to have a degree of proficiency in the use of these platforms. The rapid transitioning to online learning made it difficult to ensure all participants were fully prepared beforehand. Being forced to engage remotely, it was difficult for participants to receive the technical assistance that they would otherwise receive in person.

## Effective facilitation practices to ensure student engagement during online TBL

We adopted several different strategies to enhance the dynamism and sustain the engagement of students in online TBL sessions. Many of these revolved around consciously maintaining open forms of communication between the students and the faculty, and are as follows:

1. ***We would communicate the sequence of events and the facilitation strategy to our students and faculty at the beginning of every facilitated discussion session.*** This allowed the participants to mentally prepare for each phase of the discussion. Priority and extra time would be allocated to questions that were poorly attempted, as well as to topics corresponding to critical learning points ( Gullo, Ha and Cook, 2015).
2. ***We asked that the participants be video-enabled during the facilitated session.*** We used video as the preferred mode of communication for online TBL, because being able to see each other’s facial expressions and body language made the online discussion more dynamic for participants.
3. ***We prioritized the video discussion over private communications.*** We found that the multiple modes of communication, which are available within a single video-conferencing platform, drew attention away from the ongoing video discussion. Hence, to ensure a common learning experience, we start each facilitated discussion session with a reminder to students that private communications with the faculty were not allowed. All communications had to be made publicly.
4. ***We were able to promote peer-to-peer learning through the selective use of shared, live documents, and the ‘entire room’ chat function during the facilitated session.*** During the first phase of TBL, we used a shared, live Microsoft® Excel spreadsheet for the mTRAP process. This allowed students and faculty to see the questions and answers in real-time, which helped to promote interaction, albeit virtually. During the facilitated discussion sessions, even though we encouraged most discussions to take place over video, we allowed the students to use the ‘entire room’ chat function. This is because we recognised that the lag time from a ‘raised hand’ to being called upon in the artificially enforced, sequential nature of an online discussion was frustrating for students. Hence the use of the ‘entire room’ chat, when done sparingly, provides the students an outlet to voice their immediate thoughts, and simulates the dynamic nature of a face-to-face discussion. However, care has to be taken to ensure that the attention of the students is still focused on the ongoing video discussion. The use of the live document and the chat room enhanced the peer-to-peer learning nature of TBL in an online session, because students could read and answer the queries posted by their peers. The facilitator would then incorporate these points into the ongoing video discussion. The combination of the video-conferencing tool and the live shared document to aid the production of logical mental constructs and to enhance the learning experience, illustrate the cognitive theory of multimedia learning ( Mayer, 1997).
5. ***We held students accountable for their learning.*** To ensure that students were constantly engaged, the facilitator, using the name list at hand, would randomly call upon any student to respond or defend their team’s answer. This was one way to ensure that the students kept up with the discussion and that peer learning had occurred within the team. As we were cognizant that the students were not able to call upon their team-mates for help, we exercised patience and worked to established a secure, yet stimulating learning environment, where students felt safe to discuss questions and explore ideas ( Michaelsen and Sweet, 2008; Gullo, Ha and Cook, 2015).
6. ***The facilitation process was deliberate and we consciously practiced active listening*** ( Lane, 2008; Silberman and Biech, 2015). In general, the pace of facilitation for an online TBL session had to be slower than for a face-to-face TBL session.In a face-to-face TBL session, the facilitators could get hints from the students’ non-verbal cues on whether they were keeping up with the discussion. As this was not easily achieved in an online environment, we had to periodically check-in with the students to ensure that their queries were adequately addressed. We made sure to give students extended time to respond and acknowledged their responses before moving on.
7. ***We made sure that the students and faculty were heard.*** To increase the students’ and faculty buy-in for online TBL, we sought for feedback after each TBL session. In the subsequent TBL sessions, the facilitators will give a quick briefing on the important points that were noted. These feedback, especially during the implementation, were crucial in identifying pitfalls and strengths of the approaches employed ( Michaelsen and Sweet, 2008).
8. ***We would check in on the mental well-being of our students.*** Given that anxiety and uncertainty mount with students confined in a socially distanced learning environment, the mental well-being of students could be compromised ( Huremović, 2019), which in turn affects their learning. Studies have shown that online interactions with known contacts, may alleviate the social withdrawal symptoms of those in quarantine or forced isolation ( Pancani *et al.*, 2020; Waytz and Gray, 2018). As such, even though ensuring the mental well-being of our students is not a traditional role of the facilitator, we found ourselves in a good position to take this on because we were in frequent and regular communication with the students. In our online sessions, we would reach out to students privately, to see if they were coping well. Further information was then fed-forward to our academic coaching programme and student affairs department. A collaborative approach between facilitators/faculty and academic coaching team help to keep students motivated during these challenging times.

## Effective ways to provide support for the internet connection and technological challenges faced during online TBL classes

At Duke-NUS, we are fortunate to have a dedicated, centralized administrative team to manage the logistics of running a TBL course. This team was instrumental in the transition and implementation of online TBL. In institutions where such support is unavailable, we suggest that there should be a co-host for each facilitated discussion, who can help troubleshoot any technical issues that the students or teaching faculty may face. This will relieve the facilitator from administrative responsibilities and allow them to fully engage the students in learning. Here are the measures that we adopted to help mitigate the internet connectivity and technological issues:

• ***All participants received training in order to become proficient in the use of the online platforms.*** Prior to the online session, the administrative team carried out training sessions for all the participants; the students, facilitators and clinical teaching faculty, on the use of the various online platforms. Being familiar with the video-conferencing software helped ensure that the facilitated discussion went smoothly, and that participants were able to use the various response functions for more effective online communication.
• ***There were backup plans in place for when the online platforms were not accessible.*** Getting access to the online platforms was challenging for faculty who were participating from their host institutions that had internet separation ( MOH, 2018). The administrative team served as the conduit between the faculty who did not have access to the shared online document. Students who had connection issues were supported by the administrative team through telephone or e-mail.

## What we learnt

We found that on the whole, the facilitation practices for face-to-face and online discussions have large overlaps. However, an online facilitation requires deliberate adjustments, with the added dimensions of technical and logistical demands of online learning. These adjustments which include the training of faculty, use of multiple screens, and having a co-host, can be implemented to make facilitation easier. With careful facilitator chaperoning, by using clear and open communication, coupled with effective administrative support, we found that peer-to-peer learning could still occur through an online medium.

## Take Home Messages

Student engagement, which is key in TBL, is difficult to gauge an online format due to the inability to read non-verbal cues. Effective strategies that can be used to ensure effective peer-to-peer learning through an online medium include:

• Clear and open communication between faculty and students.
• Asking all participants to be video-enabled and for most discussion to take place in the main video-conferencing room.
• Using live, shared and interactive documents to disseminate information.
• Deliberate and slower pace of facilitation.
• Seeking feedback from all participants to improve the processes in ‘real-time’ and improve student buy-in.

Internet connectivity and technological issues can be overcome with training of participants and having backup plans in place.

## Notes On Contributors

**Peiyan Wong** is the facilitator of Phase I (preclinical phase), Office of Education, Duke-NUS Medical School, and an Assistant Professor in the Neuroscience and Behavioural Disorders Program, Duke-NUS Medical School and Department of Pharmacology, National University of Singapore; ORCID ID: https://orcid.org/0000-0003-3734-2461.

**Muhammad Raihan Jumat** is a Basic Science Education Fellow, Office of Education, Duke-NUS Medical School; ORCID ID: https://orcid.org/0000-0002-5098-6707.

**Irene Cheng Jie Lee** is an Assistant Professor, Office of Education, Duke-NUS Medical School; ORCID ID: https://orcid.org/0000-0001-6862-6438.

**Ke Xiang Foo** is an Assistant Manager of MD Programme Administrative Team, Office of Education, Duke-NUS Medical School.

**Suzanne Pei Lin Goh** is Associate Dean of Student Affairs, and facilitator for Body and Disease Course, Office of Education, Duke-NUS Medical School.

**Sashikumar Ganapathy** is an Assistant Dean, Clinical Integration, Office of Education, Duke-NUS Medical School and Deputy Head & Consultant, Emergency Medicine, KKH, Singapore; ORCID ID: https://orcid.org/0000-0001-5051-4542.

**Siang Hui Lai** is a Senior Consultant with the Department of Anatomical Pathology, Singapore General Hospital, Editor of Proceedings of Singapore Healthcare, SingHealth, Academic Vice Chair (Education), Pathology Academic Clinical Programme, SingHealth Duke-NUS AMC, and Associate Professor and Associate Dean, Office of Education, Duke-NUS Medical School.

**Nian Chih Hwang** is a Senior Consultant Anaesthesiologist with Singapore General Hospital and National Heart Centre, Singapore, and the Course Director of Body and Disease, Office of Education, Duke-NUS Medical School; ORCID ID: https://orcid.org/0000-0002-1220-345X.

## Declarations

The author has declared that there are no conflicts of interest.

## Ethics Statement

Since no personal information was collected, ethical approval was not required for this manuscript.

## External Funding

This article has not had any External Funding
