# Supplementary material for: Redesigning team-based learning facilitation for an online platform to deliver preclinical curriculum: A response to the COVID-19 pandemic
Source: MedEdPublish (2016). 2020 Jun 25;9:135. [Version 1] doi: 10.15694/mep.2020.000135.1 (PMC10702668; doi:10.15694/mep.2020.000135.1)
Supplement: Peer review history

# Reviewer report: Reviewer response for version 1

**DOI:** 10.21956/mep.20116.r31027

**Published:** 2020-07-01

**Authors:** mariam asaad amin ibrahim

**Affiliations:** 1 ain shams faculty of medicine

This review has been migrated. The reviewer awarded 5 stars out of 5 Very informative and detailed Thank you

Reviewer Expertise:

NA

No decision status is available

# Reviewer report: Reviewer response for version 1

**DOI:** 10.21956/mep.20116.r31026

**Published:** 2020-06-26

**Authors:** Subha Ramani

**Affiliations:** 1 Harvard Medical School, Brigham and Women's Hospital

This review has been migrated. The reviewer awarded 4 stars out of 5 Thank you for the opportunity to read and review this manuscript. The authors have systematically described best practices for a TBL curriculum that was delivered online due to the Covid-19 pandemic. The need for the innovation is sound, description of the intervention is clearly written such that others can adopt and adopt these best practices. The authors have also categorized the article under challenges, best practices for facilitation and how support was provided for teachers and learners.The manuscript is well written, well referenced and has clarity.I would have liked to have seen the following:(1) How faculty development was provided (2) How buy-in from teachers and learners was ensured. Was there a needs assessment, did they confer with these stakeholders?(3) A brief description of the impact of this curriculum- 2 levels of Kirkpatrick (reaction and knowledge) could have been evaluated briefly. I understand the transition was rapid, unexpected and has not been rolled out for very long.With these details and perhaps a theoretical underpinning of the benefits of TBL would make this a high quality scholarly manuscript.In its current form, it is a very good faculty development resource and informs other educators engaged in TBL courses about challenges and best practices.

Reviewer Expertise:

NA

No decision status is available

# Reviewer report: Reviewer response for version 1

**DOI:** 10.21956/mep.20116.r31025

**Published:** 2020-06-25

**Authors:** Hebat Allah A. Amin

**Affiliations:** 1 Department of Pathology, Helwan University, Faculty of Medicine

This review has been migrated. The reviewer awarded 4 stars out of 5 An important, informative experience for adopting online TBL using the official LMS. The challenge of students' engagement is the same all over the world. In our institution we adopted online TBL in a class of about 900 students. But we decided a different approach. As we stuck to our national official LMS for only the educational materials and processes with copyright requiring high degrees of security and used the commercial, free, available platforms in a portfolio of tools to provide a high degree of accessibility. We used the zoom with the breakout rooms to divide the students into teams of nine with the facilitator navigating throughout the rooms. This approach ensured the students' engagement and a close supervision. I greatly appreciate the opportunity for reading your experience. Our contribution is attached below. I hope to read more experiences from your renowned Institute. Dalia A. Gaber Mohamed Hany Shehata Hebat Allah A. Amin. (2020). MEDICAL EDUCATION ADAPTATIONS Online team‐based learning sessions as interactive methodologies during the pandemic. Medical Education. Volume 54, Issue 7. First published:26 April 2020https://doi.org/10.1111/medu.14198

Reviewer Expertise:

NA

No decision status is available

# Reviewer report: Reviewer response for version 1

**DOI:** 10.21956/mep.20116.r31024

**Published:** 2020-06-25

**Authors:** P Ravi Shankar

**Affiliations:** 1 American International Medical University

This review has been migrated. The reviewer awarded 4 stars out of 5 This is an interesting article on team-based learning and the rapid changes which had to be undertaken while shifting TBL to an online mode during the COVID-19 pandemic. The authors provide tips which would be useful to educators in other settings. Singapore being a developed high-income country some of the author’s suggestions may need to be modified while applying them to resource limited settings. The major challenge in online learning is keeping the student engaged and focused. There are a number of challenges in achieving this as has been mentioned in this and other studies. Another challenge for the teacher/facilitator during online delivery is gauging the involvement of students. TBL incorporates certain elements like the readiness assurance tests and involvement in a group which encourages attention and participation. The article will be of broad interest with the increasing adoption of TBL as a learning method.

Reviewer Expertise:

NA

No decision status is available
